# Acute Heart Failure as a First Presentation of Pheochromocytoma Complicated with “Inverted” Takotsubo Syndrome

**DOI:** 10.1155/2020/2521046

**Published:** 2020-03-17

**Authors:** Jerrold Spapen, Jeroen de Filette, Stijn Lochy, Herbert Spapen

**Affiliations:** ^1^Department of Cardiology, Universitair Ziekenhuis Brussel, Brussels, Belgium; ^2^Department of Endocrinology, Universitair Ziekenhuis Brussel, Brussels, Belgium; ^3^Department of Intensive Care Medicine, Universitair Ziekenhuis Brussel, Brussels, Belgium; ^4^Development, Ageing and Pathology Research Group, Vrije Universiteit Brussel, Brussels, Belgium

## Abstract

Takotsubo syndrome is a rare but emerging form of acute reversible myocardial injury characterized by transient systolic LV dysfunction, often related to emotional or physical stress. Pheochromocytoma is increasingly recognised as another possible trigger. Pheochromocytoma is a rare catecholamine-secreting tumour arising from chromaffin cells within the adrenal medulla or extra-adrenal paraganglia. The pathognomonic quartet of paroxysmal hypertension, palpitations, headache, and diaphoresis is rarely present, and diagnosis is often delayed. We describe a 43-year-old formerly healthy patient with an adrenal pheochromocytoma, presenting as an “inverted” takotsubo syndrome complicated with acute heart failure and pulmonary oedema.

## 1. Introduction

Takotsubo syndrome has emerged as an important cause of acute myocardial injury characterized by transient systolic left ventricular dysfunction. Multiple emotional and/or physical triggers have been identified, but the exact “stressor” remains unknown in up to 35% of patients. Pheochromocytoma is a rare neuroendocrine catecholamine-secreting tumour, originating from chromaffin cells within the adrenal medulla or extra-adrenal paraganglia. Recently, pheochromocytoma was recognised as another trigger for the syndrome. Pheochromocytoma-induced takotsubo syndrome is characterized by a more dramatic clinical presentation and higher complication rates compared to other forms. We describe a patient with an adrenal pheochromocytoma presenting as a takotsubo syndrome complicated with acute heart failure and pulmonary oedema.

## 2. Case Description

**Figure 1:**
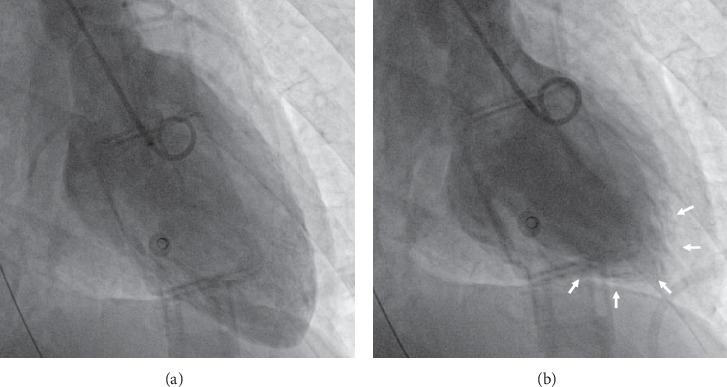
Ventriculography showing akinesia of the basal and midmyocardial segments and preserved contractility of the apex (arrows). The aspect is compatible with an inverted takotsubo pattern. (a) Diastole and (b) systole.

**Figure 2:**
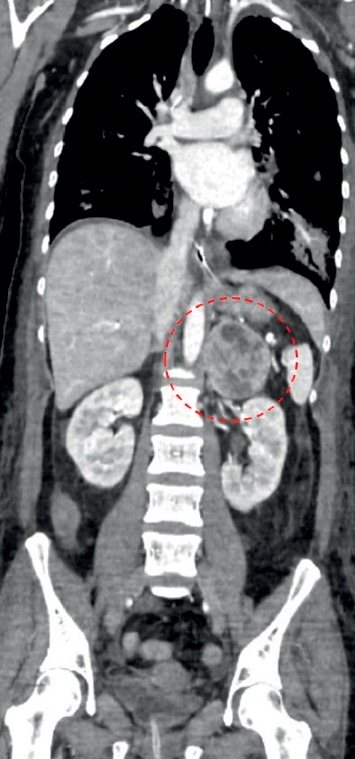
Contrast-enhanced total body CT scan revealing a left adrenal heterogeneous mass of 59 × 56 mm (red dots).

**Figure 3:**
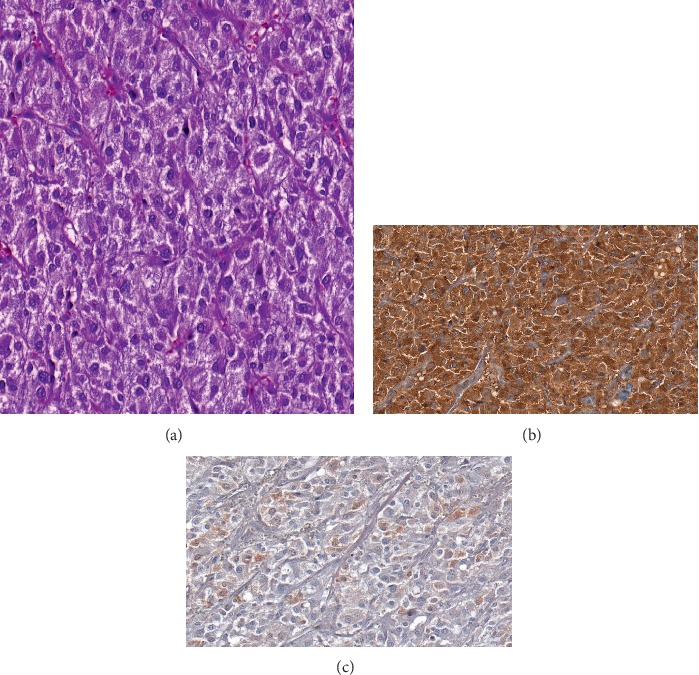
Pathological examination showing nests of polygonal cells with eosinophilic cytoplasm (a). Immunohistochemical staining is strongly positive for chromogranin A (b) and S100 (c) confirming the diagnosis of pheochromocytoma.

A 43-year-old woman without relevant medical history acutely developed nausea, severe chest pain, and dyspnoea during strenuous mountain-hiking. At a local emergency department, she was immediately intubated and initiated on intravenous nitrates because of severe arterial hypertension. At request of the family, she was transferred to our hospital. On arrival, ECG revealed an ectopic atrial rhythm, poor R-wave progression, and a prolonged corrected QT-interval. Chest X-ray showed pulmonary oedema. The troponin level was 2.42 *μ*g/L (normally <0.005), and NT-proBNP was 39,061 ng/L (normally <125). Echocardiography documented severely impaired left ventricular (LV) function (ejection fraction (EF) 35%) with basal and midsegmental akinesis and preserved contractility of the apex. Coronary angiography ruled out significant coronary disease. Ventriculography confirmed “inverted” takotsubo cardiomyopathy (Figure 1). Because of refractory high blood pressure, a diagnostic work-up was performed. Contrast-enhanced total body CT scan identified a left adrenal heterogeneous mass measuring 59 × 56 mm (Figure 2). 24-hour fractionated urinary metanephrine and normetanephrine levels were 7483 and 8895 *μ*g, respectively (normally <240 and <600). Adequate alpha-adrenergic and beta-blockade was ensured, and laparoscopic adrenalectomy was performed. Pathological examination revealed nests of polygonal cells with eosinophilic cytoplasm. Immunohistochemical staining was strongly positive for chromogranin A and S100 (Figure 3), confirming the diagnosis of pheochromocytoma. After surgery, the patient's hemodynamic condition rapidly stabilized. The LV systolic function, evaluated by repeated bedside “quick look” cardiac ultrasound, gradually improved during the following weeks. She made a further uneventful recovery and was discharged from the hospital 50 days after admission. Follow-up echocardiography at the cardiology clinic 10 weeks later showed complete normalization of LV function.

## 3. Discussion

Takotsubo syndrome (TS) is a rare but emerging form of acute reversible myocardial injury characterized by transient systolic LV dysfunction, often related to emotional or physical stress. The prevalence is estimated at 1 to 2% of patients with suspected acute coronary syndrome. TS most typically presents as pronounced regional wall motion abnormalities with akinesis of the apex and hyperkinesis of the basal segments but may appear in other variants. Recently, pheochromocytoma was recognised as another trigger for the syndrome [1, 2]. The pathognomonic quartet of paroxysmal hypertension, palpitations, headache, and diaphoresis is rarely present, and its diagnosis is often delayed. Brain haemorrhage, aortic dissection, severe LV hypertrophy, coronary vasospasm, myocardial infarction, arrhythmias, and acute heart failure with pulmonary oedema are all life-threatening complications related to high levels of circulating catecholamines, in particular norepinephrine, released by the tumour [3]. The pathogenesis of pheochromocytoma-induced TS remains elusive. A cardiocerebral relationship in TS is well established, but the exact mechanism of “neurogenic myocardial stunning” is unknown. The spillover of norepinephrine from myocardial presynaptic terminations following intense stress may induce direct cellular damage and/or epicardial and microvascular dysfunction [1, 4, 5]. Recently, persisting subclinical myocardial abnormalities (systolic/diastolic dysfunction and evidence of myocardial inflammation and fibrosis) were detected despite curative tumour resection and without biochemical disease activity. These alterations do not correlate with arterial hypertension (and subsequent pressure overload) but rather suggest direct catecholamine cardiotoxicity [6]. Pheochromocytoma-induced TS follows a remarkably more dramatic clinical course with higher complication and recurrence rates than “classic” TS. Patients are approximately 20 years younger, predominantly male (30% vs. 10.2%), and almost one-third develops a basal or “inverted” TS pattern compared to 2.2% of all TS subjects. Global forms, which are rarely seen in TS, are also more frequently reported in pheochromocytoma patients [5, 7]. By definition, TS is a self-limiting disease. Treatment is focused on supportive care and elimination of the triggering factor(s). In pheochromocytoma patients, inotropic agents are contraindicated, and beta-blockers must be combined with alpha-blocking agents to avoid a hypertensive crisis [2, 5]. Extracorporeal life support may be necessary to sustain LV systolic function while awaiting hemodynamic recovery [8]. Surgical removal of the tumour is essential. This case report highlights the importance of early suspicion and diagnostic work-up for pheochromocytoma in patients with acute heart failure of unknown origin, even in the absence of clinical evidence of catecholamine excess, especially in young patients, complicated disease course, recurrent cases, and atypical (inverted/basal or global) takotsubo subtypes.

## References

[1] Ghadri J.-R., Wittstein I. S., Prasad A. (2018). International expert consensus document on takotsubo syndrome (part I): clinical characteristics, diagnostic criteria, and pathophysiology. *European Heart Journal*.

[2] Ghadri J.-R., Wittstein I. S., Prasad A. (2018). International expert consensus document on takotsubo syndrome (part II): diagnostic workup, outcome, and management. *European Heart Journal*.

[3] Riester A., Weismann D., Quinkler M. (2015). Life-threatening events in patients with pheochromocytoma. *European Journal of Endocrinology*.

[4] Medina de Chazal H., Del Buono M. G., Keyser-Marcus L. (2018). Stress cardiomyopathy diagnosis and treatment. *Journal of the American College of Cardiology*.

[5] Y-Hassan S., Falhammar H. (2019). Pheochromocytoma- and paraganglioma-triggered takotsubo syndrome. *Endocrine*.

[6] Ferreira V. M., Marcelino M., Piechnik S. K. (2016). Pheochromocytoma is characterized by catecholamine-mediated myocarditis, focal and diffuse myocardial fibrosis, and myocardial dysfunction. *Journal of the American College of Cardiology*.

[7] Y-Hassan S. (2016). Clinical features and outcome of pheochromocytoma-induced takotsubo syndrome: analysis of 80 published cases. *The American Journal of Cardiology*.

[8] Flam B., Broomé M., Frenckner B., Bränström R., Bell M. (2015). Pheochromocytoma-Induced inverted takotsubo-like cardiomyopathy leading to cardiogenic shock successfully treated with extracorporeal membrane oxygenation. *Journal of Intensive Care Medicine*.

